# Viruses exacerbating chronic pulmonary disease: the role of immune modulation

**DOI:** 10.1186/1741-7015-10-27

**Published:** 2012-03-15

**Authors:** Aran Singanayagam, Priya V Joshi, Patrick Mallia, Sebastian L Johnston

**Affiliations:** 1 National Heart and Lung Institute, Imperial College London, Norfolk Place, London W2 1PG, UK; 2 Imperial College Healthcare NHS Trust, Praed Street, London W2 1NY, UK

**Keywords:** Asthma, cystic fibrosis, chronic obstructive pulmonary disease, respiratory viruses, rhinovirus, interferon

## Abstract

Chronic pulmonary diseases are a major cause of morbidity and mortality and their impact is expected to increase in the future. Respiratory viruses are the most common cause of acute respiratory infections and it is increasingly recognized that respiratory viruses are a major cause of acute exacerbations of chronic pulmonary diseases such as asthma, chronic obstructive pulmonary disease and cystic fibrosis. There is now increasing evidence that the host response to virus infection is dysregulated in these diseases and a better understanding of the mechanisms of abnormal immune responses has the potential to lead to the development of new therapies for virus-induced exacerbations. The aim of this article is to review the current knowledge regarding the role of viruses and immune modulation in chronic pulmonary diseases and discuss avenues for future research and therapeutic implications.

## Introduction

Chronic diseases are the leading cause of death worldwide and the third most common group of chronic diseases are chronic pulmonary diseases that account for an estimated four million deaths annually [1]. The most prevalent diseases of the respiratory tract are chronic obstructive pulmonary disease (COPD), asthma, tuberculosis and lung cancer, and the most common genetic disease is cystic fibrosis (CF). COPD is estimated to be the fourth leading cause of mortality by 2030 [2] and an estimated 300 million people suffer from asthma. COPD, asthma and CF are all chronic inflammatory conditions but their etiology and pathogenesis differ markedly. COPD and asthma are believed to be caused by exposure to relevant environmental agents (mainly cigarette smoke and aeroallergens, respectively) in patients with a susceptible genetic background, whereas CF is caused by mutations in the CF transmembrane regulator gene. The typical clinical course of these conditions is of chronic symptoms that are punctuated by periods of increased symptoms termed 'acute exacerbations'. Acute exacerbations are now recognized to be significant events in the course of the disease and have enormous implications for patients, their caregivers and for healthcare providers. Exacerbations accelerate disease progression, impair quality of life, cause significant morbidity for patients and are the major cause of mortality. In addition they are the major drivers of excess healthcare costs as they often result in unscheduled healthcare visits, treatment costs and above all hospitalizations. Therefore, preventing exacerbations is a major therapeutic goal in all three diseases and one that has not been achieved with currently available treatments.

Despite the differences between COPD, asthma and CF, all three have in common that respiratory virus infections are a major trigger of acute exacerbations. An important mechanism underlying this may be impaired host immune responses to virus infection and a better understanding of these mechanisms has the potential to lead to the development of new therapies that may be beneficial in different chronic pulmonary diseases. The aim of this article is to review the current knowledge regarding the role of viruses and host immune responses in asthma, COPD and CF, and discuss avenues for future research and therapeutic interventions.

### Induction of chronic respiratory diseases by viruses

Although this article primarily focuses on acute exacerbations of chronic respiratory diseases, virus infection has also been implicated in the induction of asthma. Asthma is strongly related to a genetic predisposition to develop allergic reactions to aeroallergens. However, not all individuals with atopy develop asthma and, therefore, it has been proposed that other environmental factors may act as 'triggers' to the development of asthma in genetically susceptible individuals. One such factor that has attracted much research interest is respiratory virus infections, in particular infection with respiratory syncytial virus (RSV). In the majority of cases RSV causes a self-limiting upper respiratory tract infection, but in infants under the age of one year it can cause a more serious infection of the lower respiratory tract - bronchiolitis - and studies have linked RSV bronchiolitis with an increased frequency of subsequent wheezing and asthma [3]. Recently, it has been reported that rhinovirus (RV) infection is also related to the development of asthma [4]. However, these studies are unable to ascertain the direction of the relationship between viral infections and asthma, that is, whether infections cause asthma or infections occur more frequently in individuals predisposed to asthma. Recent evidence has emerged supporting the later hypothesis. A study using data on hospitalization due to RSV infection for all twins born in Denmark between 1994 and 2000 found that RSV hospitalization and asthma were positively associated but that a model in which asthma 'causes' RSV hospitalization fitted the data significantly better than a model in which RSV hospitalization 'causes' asthma [5]. A study of the temporal relationship between sensitization to aeroallergens and viral wheeze showed that allergic sensitization led to an increased risk of wheezing illnesses but viral wheeze did not lead to increased risk of subsequent allergic sensitization [6]. Therefore, the link between asthma and virus infection may be due to genetically determined alterations in airway or immune responses that predispose infants both to infection and asthma, rather than virus infections causing asthma [7]. This will be discussed later in light of recent developments regarding innate immune responses in asthma but it is clear that the relationship between respiratory virus infections and the induction of asthma is complex and requires further study.

### Asthma

Asthma is the most common chronic respiratory disease affecting up to 10% of adults and 30% of children in the western world [8]. The Global Initiative for Asthma (GINA) defines asthma as 'a chronic inflammatory disorder of the airways in which many cells and cellular elements play a role. The chronic inflammation is associated with airway hyperresponsiveness that leads to recurrent episodes of wheezing, breathlessness, chest tightness, and coughing, particularly at night or in the early morning. These episodes are usually associated with widespread, but variable, airflow obstruction within the lung that is often reversible either spontaneously or with treatment'. This definition refers to the key physiological marker of asthma - reversible airflow obstruction, and the key pathological characteristic - airways inflammation. The characteristic pattern of inflammation of allergic diseases and also in asthma involves eosinophils, mast cells and T helper 2 lymphocytes (Th2) and a wide range of inflammatory mediators. Asthma exacerbations are episodes characterized by progressive increase in shortness of breath, cough, wheezing and chest tightness, or some combination of these, and increased airflow obstruction that is manifested by reductions in measurements of lung function such as peak expiratory flow (PEF). Acute exacerbations are a common occurrence in asthma and the social and economic burden of asthma exacerbations is substantial, due to both the direct costs of healthcare utilization and the indirect costs associated with lost productivity. Current therapies for asthma consist of bronchodilator and anti-inflammatory medications, the mainstay of which are inhaled β_2_-agonists and inhaled corticosteroids, respectively. These are highly effective in relieving symptoms and reduce exacerbations by approximately 50% in clinical trials [9]. However, in 'real life' surveys of asthmatics a significant proportion of patients continue to experience acute exacerbations despite therapy and, therefore, prevention/treatment of exacerbations remains a major unmet clinical need in asthma [10-12].

### Viruses and asthma exacerbations

It has long been recognized that viral respiratory tract infections are triggers for exacerbations of asthma in both adults and children but early studies reported low detection rates of viruses in asthma exacerbations casting doubt on this association. The development of highly sensitive and specific molecular diagnostic techniques using polymerase chain reaction (PCR) technology led to a reappraisal of the role of virus infections in asthma. Studies using PCR detected viruses in approximately 80% to 85% of asthma exacerbations in school-aged children and 60% to 80% of exacerbations in adults. Although respiratory virus infection can be detected in stable asthma patients detection rates are consistently lower than in exacerbated patients [13,14]. Therefore, these studies suggest that the majority of asthma exacerbations are associated with respiratory virus infections and that the low detection rates in earlier studies were a consequence of diagnostic methods with a low sensitivity. The most common viruses detected in these studies were RV. RVs are members of the picornaviridae family and are the most common cause for the common cold in both children and adults. More than 100 serotypes exist. Virus typing classified RVs into RV-A and RV-B groups based on susceptibility to anti-viral drugs and on genetic sequence similarity. More recently a newly identified group termed RV-C has been identified based purely on sequencing data [15]. Other respiratory viruses have been detected in subjects with asthma exacerbations including influenza, RSV, coronaviruses, human metapneumoviruses, parainfluenza viruses (PIV) and adenoviruses. However, in a recent study in children the only virus type significantly associated with asthma exacerbations was RV [16]. The risk of exacerbation following virus infection is influenced by other factors such as allergy and environmental pollution. Allergen sensitization, exposure to sensitizing allergens, and respiratory virus infection act in a synergistic manner to significantly increase the risk of hospitalization with acute asthma in both adults [17] and children [18]. The presence of high ambient levels of nitric oxide(NO) is also associated with an increased risk of exacerbation following RV infection [19].

### Understanding the mechanisms of virus-induced exacerbations is important for treatment strategies

Following discovery of the role of RV in asthma exacerbations research attention has focused on the mechanisms of susceptibility to virus infection in asthmatics. RV infection in healthy individuals results in a predominantly upper respiratory symptom syndrome ('common cold'), whereas in asthmatics infection results in lower respiratory symptoms and airflow obstruction ('acute exacerbation'). A study of co-habiting partners discordant for the presence of asthma demonstrated that asthmatics do not have a higher frequency of RV infections but have more severe lower respiratory symptoms and changes in airway physiology [20]. Similar results have been reported in experimental RV infection studies in asthmatics and non-asthmatic control subjects [21]. Therefore, it would appear that the consequences of virus infection in asthmatics are more severe than in non-asthmatics. Understanding the mechanisms underlying increased disease severity is crucial to developing new strategies to treat virus-induced exacerbations.

### Biology of rhinovirus infection

Most research into virus-induced asthma exacerbations has focused on RV as these are the most common viruses detected in asthma exacerbations and well-characterized models of RV infection exist both *in vitro *and *in vivo*. RVs primarily enter and replicate in epithelial cells in the respiratory tract and trigger a cascade of immune and inflammatory responses. Following viral entry into a cell, uncoating of the virus leads to the release of viral RNA that is recognized by pattern recognition receptors including toll-like receptors (TLR)-3, -7 and -8, and the cytosolic RNA helicases, retinoic acid inducible gene I (RIG-I) and melanoma differentiation-associated protein-5 (MDA-5) [22,23]. The interactions between ligand and receptor trigger signaling cascades ultimately resulting in the activation of transcription factors such as interferon regulatory factor (IRF)-3 and-7, nuclear factor-κB (NF-κB) and activating transcription factor 2 (ATF2). These activated transcription factors translocate to the nucleus and induce transcription of the type I interferons (IFN-α and -β) and pro-inflammatory cytokines including interleukin (IL)-8/CXCL8, IL-6, epithelial-derived neutrophil-activating peptide 78 (ENA-78/CXCL5) and IFN-γ-induced protein 10 kDa (IP-10/CXCL10)[24-28]. IFN-α and -β have both a direct antiviral effect through inhibition of viral replication in cells and an indirect effect through stimulation of innate and adaptive immune responses. The direct antiviral activity of type I IFNs is mediated by various mechanisms including blocking viral entry into cells, control of viral transcription, cleavage of RNA and blocking translation. These effects are mediated through the up-regulation of interferon stimulated genes (ISGs) and the production of antiviral proteins. The indirect antiviral effect is mediated through induction of natural killer cell cytotoxicity [29], up-regulation of the expression of major histocompatibility complex 1 (MHC-1) on cells and up-regulation of co-stimulatory molecules on antigen-presenting cells. Therefore, a robust interferon response is central to effective antiviral responses and resolution of virus infections. Recently a novel class of interferons termed type III interferons, or interferon-lambda (IFN-λ) has been described. The type III IFNs consist of IFN-λ1, 2, 3 (respectively, IL-29, IL-28A and IL-28B) [30]. The IFN-λs utilize a different receptor than IFN-α/β but appear to have functional similarities, however much more is known about the mechanism of action of IFN-α/β.

**Figure 1:**
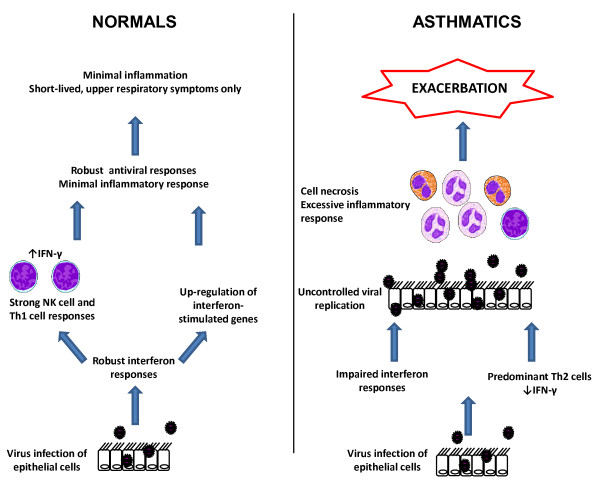
**Consequences of virus infection in healthy individuals and asthmatics**. Virus infection in non-asthmatics (left-hand panel) induces robust interferon and Th1 cell responses with rapid control of viral replication and minimal inflammation. In asthmatics (right-hand panel) impaired interferon and Th1 responses results in uncontrolled viral replication and an exaggerated inflammatory response.

The pro-inflammatory mediators and cytokines induced by RV infection lead to chemoattraction of inflammatory cells such as neutrophils, lymphocytes and eosinophils. This inflammatory response contributes to virus clearance but is also responsible for the pathology induced by RV infections. The balance between antiviral and inflammatory responses following virus infection is likely to determine the clinical outcome of the infection. An effective antiviral response rapidly controls viral replication with a minimal inflammatory response and limited clinical illness. If antiviral responses are inadequate this is likely to result in uncontrolled viral replication, greater inflammatory response and more severe clinical illness (Figure 1). The evidence that clinical illness following virus infection is more severe in asthmatics has stimulated research into possible dysregulation of antiviral and inflammatory responses in asthmatics.

### Inflammatory and immune responses to virus infection in asthma

In 2005 Wark *et al. *examined the kinetics of virus replication in bronchial epithelial cells obtained from asthmatics and healthy volunteers and reported that viral replication is increased in cells from asthmatics compared to non-asthmatic subjects [31]. This was the first report indicating that the innate immune response to virus infection may be impaired in asthma. Furthermore, the authors demonstrated that production of IFN-β was impaired in asthmatics and administration of exogenous IFN-β resulted in restoration of a normal antiviral response, and this was confirmed in a subsequent study [32]. Deficient IFN-β production by bronchial epithelial cells [33], as well as deficient IFN-α production by peripheral blood mononuclear cells [34-36] and dendritic cells [37] has also been reported in asthma. Our group has also shown that IFN-α and IFN-β production by alveolar macrophages is impaired in asthmatics (manuscript submitted). Furthermore, deficient IFN-λ production by epithelial cells and alveolar macrophages in asthmatics has also been reported and related to clinical outcomes following experimental RV [38]. However, other groups have not reported deficient IFN induction in epithelial cells from asthmatics [39,40]. In experimental RV infections virus loads were higher and virus shedding prolonged in asthmatics but this was not statistically significant [21,41]. Therefore, although interferon deficiency is an exciting new mechanism underlying increased severity of virus infection in asthma it has not been conclusively demonstrated to occur in all asthmatic subjects studied. The studies in question were small with different experimental conditions such as cell culture techniques and virus dose. It is also possible that interferon deficiency occurs in some asthmatics only and it may also be related to disease severity, disease control or degree of atopy. Further studies with more subjects and careful patient selection and characterization are required to provide answers to these ongoing research questions.

If interferon production in response to virus infection is impaired in asthmatics what are the possible molecular mechanisms underlying this? The discovery that IFN-α, IFN-β and IFN-λ are all deficient suggests that it is not a genetic defect as IFN-α and IFN-β are on different genetic loci than IFN-λ. A key family of proteins regulating both interferon production and allergic inflammation are the suppressor of cytokine signaling family (SOCS), and one member of this family, SOCS1, is a potent negative regulator of type I and type II interferons and of Th2 inflammation [42,43]. SOCS1 is induced by type II cytokines such as IL-13 [44] and, therefore, persistent Th2 inflammation may result in chronic up-regulation of SOCS1 and impaired interferon responses, but this hypothesis requires further investigation.

### Inflammatory responses to virus infection in asthma

*In vitro *infection of airway epithelial cells with RV induces the production of inflammatory mediators and this has also been reported *in vivo *in both experimental and naturally-acquired viral infections. Chemokines and cytokines such as IL-8, IL-6 and regulated on activation, normal T-cell expressed and secreted (RANTES) have been detected during virus infections in asthmatic patients [45-49]. However it remains unclear whether the inflammatory response following virus infection differs quantitatively or qualitatively in asthmatics. One experimental RV infection study reported increased nasal lavage levels of IL-8 and IL-1β in asthmatics [46] but not in control subjects; however, another study reported no differences in IL-6, IL-8, IL-11 and granulocyte-monocyte-colony stimulating factor (GM-CSF) levels in either nasal lavage or sputum between asthmatics and non-asthmatics [45]. Increased sputum levels of IL-10 but not RANTES or IL-8 have been reported in asthmatics [48]. These conflicting results highlight the need for further studies evaluating the inflammatory profile (preferably in the lower airway) in well-characterized patients and non-asthmatic controls following virus infection.

Many of the inflammatory mediators produced are chemoattractants and, therefore, following virus infection inflammatory cells are recruited to the lungs. A number of different inflammatory cells have been identified in both naturally-occurring and experimental virus infections in asthma. Although stable asthma is characterized by eosinophilic inflammation, a number of studies have identified neutrophils as the key inflammatory cell in virus-induced asthma exacerbations [21,50-52]. Neutrophils release bioactive mediators such as the protease neutrophil elastase that have effects such as stimulation of mucous production and, therefore, are likely key contributors to the pathogenesis of asthma exacerbations. Another key cell involved in immune and inflammatory responses in the lungs is the macrophage. There is evidence that RVs can infect macrophages and that in asthmatics macrophage responses to virus infection are altered. Our group has reported that RVs infect macrophages and induce TNF-α production [53] and that production of the cytokine IL-15, that plays a key role in linking innate and adaptive antiviral immune responses and promoting T cell anti-viral immune responses, is impaired in asthmatics [53]. As described previously, macrophage production of IFNs in response to virus infection is also impaired in asthma [38]. Therefore, there is evidence of impaired antiviral responses in asthmatics in macrophages as well as respiratory epithelial cells.

Increased lymphocyte numbers in bronchoalveolar lavage (BAL) and bronchial biopsies in experimental RV infection in asthmatics has been reported, with increases in CD4+, CD8+ and NK cells [21,54]. Abnormalities of the acquired immune system in stable asthma have been well described with skewing of acquired immune responses towards a Th2 profile. As robust antiviral responses require an adequate Th1 response it is possible that in diseases such as asthma with predominant Th2 cells antiviral immunity is impaired. Impaired levels of the Th1 cytokines IL-12, -15, -18 and IFN-γ have all been reported in asthma [21,55-57]. In human experimental RV infection lower respiratory symptoms, bronchial hyperreactivity, reductions in blood total and CD8+ lymphocytes and virus load are related to deficient IFN-γ, IL-12 or IL-15 responses and to augmented IL-4, IL-5, and IL-13 responses [21,55]. Sputum IFN-γ/IL-5 messenger RNA ratio following virus infection is inversely related to both peak cold symptoms and the time to viral clearance [58]. Therefore, augmented Th2 and deficient Th1 immune responses are associated with greater clinical illness following RV in asthma.

### Induction of asthma

The identification of impaired innate immunity in asthma suggests a possible mechanism not only for virus-induced asthma exacerbations but also for the link between respiratory virus infections and the subsequent development of asthma. It is possible that infants who will develop asthma in later life have impaired immune responses from birth and, therefore, are more likely to develop more severe disease manifestations (for example, bronchiolitis) following respiratory virus infection. Most studies to date have focused on the role of the acquired immune system and identified reduced IFN-γ production as a significant risk factor both for subsequent wheezing illness and allergic sensitization [59-61]. No studies have investigated innate immune responses in infants prior to the development of symptomatic asthma but impaired IFN-α production has been reported in older children with atopic asthma [35].

In conclusion there is evidence that both innate and acquired immune responses in asthmatics are impaired and this may be a key mechanism underlying virus-induced asthma exacerbations and the link between virus infections and the subsequent development of asthma in infants. Further studies are needed to determine whether these deficiencies are common to all asthmatics, whether they represent a specific asthma phenotype and how they relate to conventional measures of asthma control. Another important research question is whether new interventions targeting the interferon pathways can prevent asthma exacerbations and even potentially prevent the development of asthma in infants.

### Chronic Obstructive Pulmonary Disease

Chronic obstructive pulmonary disease (COPD) is the most common chronic respiratory condition in adults. The Global Initiative for Obstructive Lung Disease (GOLD), a collaboration between the World Health Organization and the National Heart Lung and Blood Institute, defines COPD as 'a preventable and treatable disease with some significant extrapulmonary effects that may contribute to the severity in individual patients. Its pulmonary component is characterized by airflow limitation that is not fully reversible. The airflow limitation is usually progressive and associated with an abnormal inflammatory response of the lung to noxious inhaled particles or gases' [62]. The main etiological agents linked with COPD are cigarette smoking and biomass exposure and the inflammatory response consists of neutrophils, macrophages and CD8+ T cells and, therefore, differs from the allergic inflammation seen in asthma. Pulmonary inflammation is further amplified by oxidative stress and excess proteases released by inflammatory cells recruited to the lung. As in asthma, acute exacerbations are a common occurrence in COPD and become more frequent as the disease progresses [63]. Exacerbations are a major cause of morbidity, mortality and healthcare costs and accelerate decline in lung function [64] and quality of life [65] in COPD patients. Historically, bacterial infections have been considered the predominant infectious etiology, however epidemiological data showing a greater frequency of exacerbations in the winter months [66] and frequent coryzal symptoms preceding exacerbations suggest a causal role for viruses [67]. Older studies using cell culture and serologic diagnostic tests detected viral infection in only approximately 10% to 20% of exacerbations [68,69]. However, these diagnostic methods have low sensitivity for virus detection especially for RVs that are the most common cause of upper respiratory tract infections. More recent studies using modern PCR-based techniques have allowed a re-evaluation of the importance of viruses in COPD exacerbations and these studies have shown the presence of viruses in 47% to 56% of exacerbations [70-73]. A recent systematic review evaluated weighted mean prevalence of respiratory viruses detected by PCR in patients with acute exacerbations of COPD. Eight studies were included with an overall prevalence of 34.1%, with picornaviruses including RVs being the most frequently detected pathogen, followed by influenza, parainfluenza, RSV and adenoviruses [74]. Although these studies have higher detection rates they are likely to have underestimated the role of viral infections in COPD exacerbation as they evaluated patients at the time of presentation to healthcare services which often occurs considerably later than the onset of exacerbation and by which time virus may no longer be detectable.

### Experimental infection studies in COPD

Although viruses are frequently detected in COPD exacerbations, their presence during exacerbations does not prove a definite causative role. Experimental infection using RV provides a novel tool for investigating relationships between virus infection and exacerbations. Such studies have been previously conducted in asthma and yielded important insights into the mechanisms linking virus infection to exacerbations in asthma. A recent study from our group reported the first experimental RV infection study in COPD [75]. COPD patients and non-obstructed controls were infected with RV with sequential measurement of symptoms, lung function, inflammatory markers and virus load. Following RV infection, COPD subjects developed symptomatic colds followed by the typical lower respiratory symptoms of an acute exacerbation. Symptoms were accompanied by objective evidence of airflow limitation and airways inflammation and inflammatory markers correlated with virus load. Virus was detected in airway samples prior to the onset of symptoms and viral clearance was followed by symptom resolution and return of inflammatory markers to baseline levels. Therefore, this study directly links respiratory virus infection to lower respiratory symptoms, airflow obstruction and airways inflammation in COPD and provides novel evidence supporting a causative role for RV infection in COPD exacerbations.

### Mechanisms of virus-induced COPD exacerbations

Much less is known regarding mechanisms of virus-induced exacerbations in COPD compared to asthma. In the experimental infection study, symptoms, airflow obstruction and airways inflammation were more severe in the COPD subjects compared to non-obstructed controls [75]. Therefore, as is the case in asthma it would appear that clinical illness following RV infection is more severe in COPD subjects, but the mechanisms underlying this are poorly understood. COPD exacerbations are associated with increased levels of inflammatory mediators including tumor necrosis factor-alpha (TNF-α) [76], IL-8 [76,77], IL-6 [78], and leukotriene B4 [79] and inflammatory cells such as neutrophils [70,77] and eosinophils [70]. However, few studies have examined the inflammatory response specific to virus-induced exacerbations. Virus infection has been associated with high levels of IL-6 [80,81] and IP-10 [82,83] and Papi *et al. *reported that elevated sputum eosinophils were only seen in exacerbations in which a virus was present [70]. Others have reported that the presence of RV is not associated with significant airway inflammation [84] and that only exacerbations associated with purulent sputum (presumed bacterial infection) are associated with airways inflammation [79]. From the data available no clear conclusions can be drawn regarding the inflammatory response to virus infection in COPD and there are no studies comparing the effects of naturally-occurring virus infections in COPD patients and non-COPD controls.

There is evidence from animal models that the inflammatory response to virus infection may be exaggerated in COPD. In a mouse model of COPD utilizing intranasal administration of lipopolysaccharide and elastase, infection with RV resulted in increased levels of TNF-α and IL-13 compared to control mice [85]. This was accompanied by increased airway hyper-responsiveness and increased mucus production. Similarly, in the human COPD RV challenge study, increased levels of IL-8 and neutrophil elastase were reported in COPD subjects when compared to non-obstructed controls [75]. These studies suggest that COPD is associated with an exaggerated inflammatory response to viral infection and this may explain the increased severity and duration of symptoms seen in these patients.

*In vitro *studies have shown that cigarette smoke impairs release of IFN-β and IFN-α [86]. BAL cells from COPD patients infected *ex vivo *with RV demonstrated deficient induction of IFN-β with similar trends for deficient induction of IFNs-α and -λ, associated with deficiency of the interferon stimulated gene CXCL10 [75]. Similar findings have been reported in a mouse model where persistence of RV, increased airways inflammation and deficient induction of IFNs-α, -β and -γ were reported in COPD mice compared to controls [85]. However *in vitro *RV infection of epithelial cells from COPD patients resulted in higher virus load and increased inflammatory mediators, but no differences in interferon production compared to cells from control subjects [87]. Further studies examining the role of interferon deficiency in viral exacerbations are required as this may lead to potential future therapeutic application of interferon therapy in reducing exacerbation severity in COPD. RVs bind to cells via intercellular adhesion molecule-1 (ICAM-1, major group RVs) or members of the low-density lipoprotein receptor family (minor group RVs). ICAM-1 is upregulated on the bronchial epithelium of patients with COPD [87,88] and, therefore, it is possible that increased ICAM-1 levels may permit greater virus binding and increased viral entry into epithelial cells in COPD patients.

### Virus infection and stable COPD

The majority of studies have detected viruses at a greater frequency during acute exacerbations compared to the stable state. One study indicated that RSV is detected in nasal lavage at a similar frequency of around 25% in the stable state and during exacerbations [67]. This was followed by a similar study reporting detection of RSV in about 30% of sputum samples, with detection being related to greater airway inflammation and to a faster decline in lung function [89]. However, other studies have not reported increased RSV detection in stable COPD [70,71]. A study comparing virus loads between infants with acute respiratory infections and adult COPD patients found that virus loads were 2000-fold higher in the infants, suggesting low-grade virus infection in COPD [90]. The disparity between these findings is likely to be due to a combination of factors including differing sensitivity of the PCR techniques used, differences in severity of COPD patients included or differences in the populations studied [91].

Latent infection by adenovirus has also been proposed to be involved in the pathogenesis of COPD. Lung tissue from COPD patients has been demonstrated to carry more group C adenoviral DNA than matched non-obstructed smokers [92]. Latent adenoviral infection in combination with cigarette smoke exposure in a guinea pig model caused an increase in lung volumes, airspace volume and reduced surface to volume ratio compared to smoke exposure alone [93]. Additionally, adenovirus detection has been shown to be similar in exacerbated and stable COPD patients [94]. Some authors have postulated that the presence of RSV and adenovirus in stable COPD may contribute to the pathogenesis of the disease as there are some common pathologic features between respiratory viral infection and COPD including a predominance of CD8+ T lymphocytes. However, this remains a largely unproven hypothesis.

### Cystic Fibrosis

Cystic fibrosis (CF) is an autosomal recessive disease caused by mutations in the gene for the cystic fibrosis transmembrane regulator (CFTR) protein. Defective CFTR function leads to abnormal transport of chloride and sodium across the pulmonary epithelium, resulting in viscous secretions in the lungs, recurrent bacterial infections and progressive loss of lung function. Pulmonary involvement is the most common manifestation of the disease and respiratory failure the most common cause of death. Respiratory infections are the leading cause of morbidity, decline in lung function and hospitalizations due to acute exacerbations. The major cause of infectious complications in CF has always been considered to be bacterial infection, with *Pseudomonas aeruginosa *the most common organism detected. There has been relatively little research on the role of virus infections in CF but recent studies have suggested that viruses have a significant impact on the CF patient.

### Viruses and CF exacerbations

The role of respiratory viruses in CF exacerbations is likely to have been under-appreciated in the past because older studies investigated only one virus type and the detection methods used were not sufficiently sensitive. Newer PCR techniques have helped to improve detection and it is now becoming clear that viruses are implicated in exacerbations in CF. Previous studies using serology, culture and immunoflourescence detected viruses in 10% to 28% of exacerbations in CF patients [95-98]. In contrast, studies using PCR for virus detection have reported detection rates of 50% to 60% [99-101]. A number of different viruses have been detected in CF patients with the most common being RVs, influenza and RSV. The incidence of viral infections in children with CF is not elevated in comparison to healthy children but the severity of clinical illness associated with infection is greater [102]. Viral infections are associated with deterioration in lung function and more severe clinical illness indicating that they contribute to disease progression thus demonstrating the clinical importance of research within this field [100,103].

### Mechanisms of virus infection in CF

The mechanisms of viral-induced CF exacerbations and increased clinical illness are poorly understood with conflicting results from published studies. Some authors have reported increased production of pro-inflammatory cytokines and chemokines by epithelial cells obtained from CF patients compared to healthy controls [102,104]. However, others have failed to detect any differences in cytokine production between CF and normal cells [105,106]. These differences may be due to different viruses used (RV, RSV, PIV) and differences in cell culture techniques, but it remains unclear whether the CF epithelium is intrinsically pro-inflammatory in response to virus infection. Another mechanism that has been postulated is a deficiency in antiviral innate immune responses in CF cells. Increased replication following PIV infection of CF cells has been reported and this was corrected by administration of IFN-α [102]. IFN responses were not impaired but induction of nitric oxide synthase 2 (NOS2) was impaired in CF. NOS2 is required for production of NO that has potent antiviral effects and, therefore, impaired NO synthesis may be one mechanism of impaired antiviral host responses in CF. Our group has reported reduced IFN-β and IFN-λ production and reduced ISGs in CF epithelial cells [107] and, therefore, IFN deficiency may be relevant to CF as well as in asthma and COPD. Holtzman has proposed that 'hypersusceptibility' to virus infection, via defective interferon pathways, is a unifying pathway in asthma, COPD and now CF [108].

### Bacteria-virus interactions in pulmonary disease

Both bacterial and virus infections are common in CF and COPD and, therefore, co-infections are likely to be common. There is now increasing evidence that both viral and bacterial infections can modulate host immune responses and increase susceptibility to subsequent infection. There is abundant evidence from both human studies and animal models that influenza infection impairs antibacterial immunity and this can result in secondary bacterial pneumonia [109,110]. However, much less is known regarding the effect of other respiratory viruses, such as RVs, on susceptibility to bacterial infection. *In vitro *studies have reported that RV infection increases bacterial adhesion to epithelial cells [111-113] and impairs macrophage immune responses to bacterial products [114]. We have found that experimental RV infection in COPD is followed by secondary bacterial infection in 60% of patients and this is related to deficiency of the antimicrobial peptides elafin and secretory leukoprotease inhibitor (SLPI) (submitted manuscript). There are also studies indicating that virus-bacteria interactions influence host immune responses in CF. Chattoraj *et al. *reported that RV infection of CF cells liberates planktonic bacteria from biofilm [115]. Planktonic bacteria express virulence factors and stimulate inflammatory responses more readily compared to biofilm bacteria and this was manifested by increased cytokine responses. Evidence is also emerging that bacterial infection can increase susceptibility to viral infection. Infection of epithelial cells by *Haemophilus influenzae *(a common organism in COPD) increases susceptibility to infection by RV, possibly by up-regulation of ICAM-1 [116]. CF cells infected with mucoid *P.aeruginosa *and then with RV produced less IFN and viral loads were higher compared to cells infected with the RV alone [117]. This effect was not seen in normal epithelial cells infected with *Pseudomonas *and was related to the inhibition of Akt phosphorylation and IRF-3 activation - both pre-requisites for the IFN response to RV infection.

It is widely acknowledged that the main infectious cause of asthma exacerbations is virus infection and it is believed that bacteria play only a minor role. However, a recent study using culture-independent molecular methods for bacterial detection reported that the bacterial flora in the airways of asthmatics is closer to that of COPD patients than of non-asthmatics [118]. The role of bacteria in asthma exacerbations needs to be revisited as virus-bacterial interactions may play a role in the pathogenesis of asthma exacerbations. This is a fertile area for further research.

Our knowledge of the interactions between respiratory viruses and bacteria, and how these influence host immune responses in pulmonary diseases, is still at an early stage. Further research is required to understand better these complex relationships and to explore the implications they may have for the development of new therapies.

## Conclusions

There is now convincing data implicating respiratory viruses as a major cause of acute exacerbations in asthma, COPD and CF. In all these conditions there is evidence that host immune responses to virus infection are impaired, but whether this occurs through a common mechanism, or whether mechanisms differ between the different diseases is unclear. Further research is needed to elucidate the exact mechanisms of increased susceptibility to virus infection in pulmonary diseases, the interactions between viruses and bacteria and how these impact on host immune responses. A better understanding of these mechanisms has the potential to lead to the development of novel therapies that will reduce the impact of acute exacerbations in chronic pulmonary diseases.

## List of abbreviations

ATF: activating transcription factor; BAL: bronchoalveolar lavage; CF: cystic fibrosis; CFTR: cystic fibrosis transmembrane regulator; COPD: chronic obstructive pulmonary disease; ENA-78: epithelial-derived neutrophil-activating peptide 78; ICAM-1: intercellular adhesion molecule; IFN-α: interferon-alpha; IFN-β: interferon-beta; IFN-λ: interferon-lambda; IFN-γ: interferon-gamma; IL: interleukin; IP-10: IFN-γ-induced protein-10; IRF: interferon regulatory factor; ISG: interferon stimulated genes; MDA-5: melanoma differentiation-associated protein-5; NF-κB: nuclear factor-kappa B; NO: nitric oxide; NOS2: nitric oxide synthase 2; PCR: polymerase chain reaction; PEF: peak expiratory flow; PIV: parainfluenza virus; RANTES: regulated on activation: normal T-cell expressed and secreted; RIG-I: retinoic acid inducible gene I; RSV: respiratory syncytial virus; RV: rhinovirus; SLPI: secretory leukoprotease inhibitor; SOCS: suppressor of cytokine signaling family; Th1/2: T helper 1/2; TLR: toll-like receptors; TNF-α: tumor necrosis factor-alpha -1.

## Competing interests

SLJ has patents on the use of interferons as a treatment for asthma exacerbations and COPD exacerbations and share options in Synairgen. AS, PVJ, and PM declare that they have no competing interests.

## Authors' contributions

All the authors contributed equally to writing the manuscript. All authors read and approved the final manuscript.

## Authors' information

SLJ heads a group working on mechanisms of asthma and chronic obstructive pulmonary disease and the role of respiratory viral infections in these diseases, with a particular interest in acute exacerbations and the role of rhinovirus infections. PM and AS work in his group with a particular focus on human and mouse models of virus infection in COPD. PVJ works in the Department of Respiratory Medicine at Imperial College NHS Healthcare Trust.

## Pre-publication history

The pre-publication history for this paper can be accessed here:

http://www.biomedcentral.com/1741-7015/10/27/prepub
